# Role of Complement Components in Asthma: A Systematic Review

**DOI:** 10.3390/jcm13113044

**Published:** 2024-05-22

**Authors:** Ilona Tornyi, Ildikó Horváth

**Affiliations:** 1 Department of Pulmonology, Faculty of Medicine, University of Debrecen, 4032 Debrecen, Hungary; tornyi.ilona@med.unideb.hu; 2 National Koranyi Institute of Pulmonology, 1121 Budapest, Hungary

**Keywords:** asthma, innate immunity, complement components, complement regulators, biomarker

## Abstract

**Background:** Asthma is a chronic inflammatory airway disease characterized by recurrent symptoms in response to a wide range of external stimuli, including allergens, viral infections, and air pollution together with internal host-derived danger signals. The disease is traditionally associated with adaptive immune responses; recent research emphasizes the critical role of innate immunity in its pathogenesis. The complement system, activated as part of the defense mechanisms, plays a crucial role in bridging innate to adaptive immunity. While experimental models demonstrate complement cascade activation in asthma, human studies remain limited. **Methods:** This systematic review summarizes existing literature on the complement system in asthma patients, gathering data from PubMed, Web of Science, Scopus, and Google Scholar. The protocol was registered in the OSF. **Results:** Out of 482 initially identified articles, only 24 met the eligibility criteria, revealing disparities in sample origin, methodologies, and populations. Despite observed heterogeneity, a consistent result was found in the elevation of complement regulatory proteins, such as complement Factor H, in samples from patients with asthma compared to those from healthy subjects. **Conclusions:** The increased level of regulatory proteins, such as Factor H and I highlight that these may influence asthma pathophysiology. The role of complement factors as potential biomarkers of asthma activity and severity needs further evaluation.

## 1. Introduction

Asthma is a chronic respiratory disorder, characterized by chronic airway inflammation, airway hyperreactivity, and remodeling. It is a complex disease, in which the inter-play between genetic and environmental factors has profound influence not only in its development, but also in its natural history, treatment response, and comorbidities. Environmental factors such as allergens, smoke and other outdoor and indoor air pollutants, viruses, and bacteria are among the most important risk factors for the development of asthma in genetically susceptible individuals [1]. Besides the complex genetic back-ground, aberrant immune maturation in early life resulting in dysfunctioning defense pathways, including inappropriate type 2 immune reactions to different stimuli, plays an important role in the pathomechanism [2].

Treatment recommendations for asthma typically depend on the severity of the condition and may vary from person to person. Inhaled corticosteroids serve as the primary and most effective treatment [3,4]. There have been numerous other attempts, with varying degrees of success, to treat asthma. Leukotriene modifiers can serve as an alternative treatment for adults with mild or aspirin-sensitive asthma [5]. However, they are less effective as monotherapy but, as an add-on therapy, they could reduce the dose of corticosteroids in moderate and severe asthma [6]. Long-acting ß2-agonists may be beneficial in reducing the frequency of exacerbations [7]; however, since these medications do not seem to affect airway inflammation, these should only be used in combination with corticosteroids, not as monotherapy [6]. For patients with acute severe asthma resistant to standard treatments, different drugs have been tried with controversial results [8]. For example, ketamine has been used both in children and adults; however, the existing evidence does not strongly support its use. Moreover, there are indications of potential adverse effects linked to its administration [9]. 

Allergens, viruses, smoking, and air pollutants as danger signals trigger the activation of different defense pathways in the airways to eliminate the “invader”. Activation of the innate immune system provides rapid, nonspecific protection against a wide range of triggers, but lacks the specificity and memory. It encompasses various cellular and molecular mechanisms, including epithelial barriers, pattern recognition receptors, phagocytic cells, proinflammatory cytokines, and the complement system.

Epithelial cells, covered by mucus, serve as the first mechanical defense against environmental factors. Airway mucus contains several components that contribute to host defense, including mucoglycoproteins, proteoglycans, immunoglobulin A (IgA), lactoferrin, lysozyme, and defensin [10]. On the surface of epithelial cells pattern recognition receptors can be found in order to detect cellular stress factors such as damage or pathogen signals. Eosinophils activated by IL-4, IL-5, IgA, and IgG, damage and pathogen activation signals, produce growth factors and fibrogenic modifiers that contribute to tissue remodeling through increased collagen, glycosaminoglycans production, and proliferation of fibroblasts. Activated eosinophils produce IL-4 and IL-13 that stimulate IgE production [11] and B cell proliferation [12]. Depending on the type of stimuli that eosinophils receive, they produce different chemokines that attract T helper-1 (Th1) or T helper-2 (Th2) cells [13]. Indoleamine 2,3-dioxygenase, also produced by eosinophils (and macrophages [14], fibro-blasts [15], endothelial cells [16], and dendritic cells [17]), provides a negative feed-back to Th1 cells, and upregulates Th2 cells [18], thus interrupts the balance between Th1/Th2 cells produced by cytokines. These cytokines (Th1: INF-γ; Th2: IL-4, IL-5, IL-13) play an important role in the development and evolution of asthma [19]. Th2 lymphocytes regulate the increased responsiveness of the tracheobronchial tree in asthma by secreting cytokines that mediate the inflammatory immune response, particularly in allergic asthma where IgE production by B cells is enhanced [20].

The complement system is a crucial component of the ancient first-line danger-sensing and defense innate immune system [21]. Therefore, it is plausible that complement activation occurs and has a role in asthmatic airways. It consists of more than forty soluble proteins in blood and other body fluids. These proteins circulate in an inactive zymogen form and are organized into a complex network cascade that can be activated directly by pathogens or indirectly by pathogen-bound antibodies. There are three ways of activation: the classical pathway, the lectin pathway, and the alternative pathway [22]. The classical pathway can be activated via IgM, IgG, or pattern recognition molecules (pentraxins, microbial or apoptotic cells) through C1q. C1s, as a part of C1 complex, cleaves C4 into C4a and C4b. Then C4b cleaves C2 into C2a and C2b. These parts are assembled into the C3 convertase C4b2b [23]. In the lectin pathway, mannose-binding lectin and ficolin recognize a carbohydrate pattern and assemble with mannose-binding lectin-associated serine proteases (MASPs) to initiate this pathway. MASP-2 cleaves C2 and C4 to generate C3. MASP-1 could cleave only C2, that contributes to the previously initiated lectin pathway [24]. The alternative pathway spontaneously activated by C3, controlled by Factor B and Factor D, generates C3bBb (also called C3 convertase). All C3 convertases cleave C3 into an inflammatory mediator C3a and opsonin C3b. With the help of cleaved C3b, a C3 convertase could gain a new function as a C5 convertase (C4b2b3b or C3bBb3b). Factor H regulates the alternative pathway through C5 convertase dissociation. It acts as a soluble inhibitor of C3 convertase with binding to C3b. With binding to C3b, Factor H blocks Factor B and Factor D to interact with C3b, thus forming the C3 convertase. As a cofactor of Factor I, Factor H promote C3 convertase (C3bBb) dissociation. Factor I can control all three pathways by degrading cleaved C3b and C4b in cooperation with different cofactors (Factor H, CR1, CD46, C4b-binding protein) [24]. Factor I circulates in an inhibited form; it becomes active only in the presence of its cofactors. Factor I, with its protease activity, cleaves C3b into fragments such as iC3b, which factor B is no longer able to bind. These functions prevent complement activation on host cells. Spontaneous hydrolysis of C3 exposes binding sites for Factor B. When bound to C3, Factor B activates Factor D, which cleaves this complex into C3Bb, what is a soluble phase C3 convertase enzyme complex. C3Bb cleaves C3 into bioactive fragments such as C3a and C3b that leads to further complement activation [23,25].

C5 could be cleaved into C5a and C5b. Finally, C5b could form a complex with C6, C7, and C8, and inserts into a cell membrane, thus arranging C9 proteins and forming a pore-forming terminal complex [21].

One of the key functions of the complement system is the elimination of invaders via opsonization. Besides surveillance, this system plays a role in the clearance of apoptotic cells [26], maintenance of homeostasis by promoting tissue repairing [27], local enhancement of epithelial cell defense [28], cooperation with Toll-like receptors in proinflammatory cytokines production [29], complementing B-cell humoral immunity [30], and regulation of cellular immunity T-cells [31]. It also plays an important role in hyperinflammation and thrombotic microangiopathies leading to coagulopathy in response to infections as highlighted in the pathomechanism of coronavirus disease 2019 (COVID-19) [32]. Through these mechanisms, the complement axis plays a crucial role in multiorgan inflammatory dysfunctions in acute COVID-19 and consequently likely influences the development of different co-morbidity clusters in post-COVID-19 syndrome [33].

As a part of innate immunity, complement factors contribute directly and indirectly to the development of asthma. This occurs directly, as part of the local inflammation, through eosinophil activation via eosinophil surface receptors for C3a and C5a that, in the case of C3a or C5a binding, promote eosinophil activation [34]. This occurs indirectly through the adaptive immune system, by modulating the activity of other immune cells and cytokines involved in the disease process, and the function loss of inflamed airway epithelium [35].

Mice have been extensively studied as valuable experimental models for mimicking asthma phenotypes [36]. In a severe asthma mouse model, Lajoie et al. have highlighted a significant involvement of C3 and C5 in regulating the IL-23-TH17 axis [37]. Mizutani et al. demonstrated that C3a plays a crucial role in the development of the late asthmatic response and airway hyper-responsiveness, possibly through the recruitment of neutrophils and induction of IL-1β production [38]. In an allergic asthma mouse model, following the ovalbumin antigen challenge, an upregulation of complement components such as C1q and C3 was observed [39]. All of these studies suggest the activation and potential regulatory role of the complement cascade in asthma.

There are, however, limitations in using animal models in asthma research and extrapolating data to humans. Mouse models do not spontaneously develop asthma and typically consist of adult animals with normally developed immune systems. Furthermore, the morphology and arrangement of the bronchial tree significantly impact allergen penetration into the lungs, while the size of the animal greatly influences lung function [36]. Although many aspects of asthma can be investigated through animal models; no single murine model can fully replicate all the complexities of human asthmatic disease. Therefore, to understand the role of complements in patients with asthma, data from samples derived from patients are essential.

The aim of the current systematic review is to evaluate the available evidence related to complement system measurements in biological samples of asthma patients.

## 2. Materials and Methods

This systematic review of the available literature addressing asthma and the complement system was conducted according to the Preferred Reporting Items for Systematic Reviews and Meta-Analyses (PRISMA) statement [40] and registered into Open Science Framework (doi: 10.17605/OSF.IO/MT6HS).

### 2.1. Searching Strategy and Inclusion/Exclusion Criteria

We performed a systematic literature review to identify relevant publications using electronic databases such as PubMed, Web of Science, Scopus, and Google Scholar up to 1 April 2023. The searching algorithm was based on a primary search keyword “asthma” AND secondary keywords such as “Complement component(s)” OR “Complement system components” OR “Complement complex” OR “Complement factor” OR “Complement C1/C1q/C2/C3/C3a/C4/C4a/C5/C5a/C6/C7/C8/C9” OR “sC5b-9” OR “C5b-9”. Publications were searched with the keywords combination in the PubMed database for the “Title/abstract” search, in the Web at Science database for the “Topic [title, abstract, author key-words, and keywords plus]” search, and in the Scopus database for the “Abstract” search. The authors performed a manual search at Google Scholar for articles, with the above-mentioned keywords, without PubMed ID.

Studies were excluded if the study in the following instances: language was not in English; did not present original data (review, book chapter, meta-analysis); was not a full text (abstract, or article was not available); was not performed on human subjects (animal studies, in vitro studies).

The study selection criteria are presented in Table 1.

### 2.2. Quality Assessment

In the evaluation of studies using the Newcastle–Ottawa Scale (NOS), a comprehensive framework was employed to assess the methodological quality. The NOS addressed three pivotal domains: the selection of participants, comparability between study groups, and the evaluation of outcomes. Each domain was assigned a maximum score, and studies were categorized into low, moderate, or high quality based on their cumulative scores, with thresholds of 0–3, 4–6, and 7–9 points, respectively [41].

The literature searching and quality assessment were carried out by two authors.

There was no possibility to perform a meta-analysis due to the heterogeneity of measurement methods and sample origin.

## 3. Results

**Figure 1:**
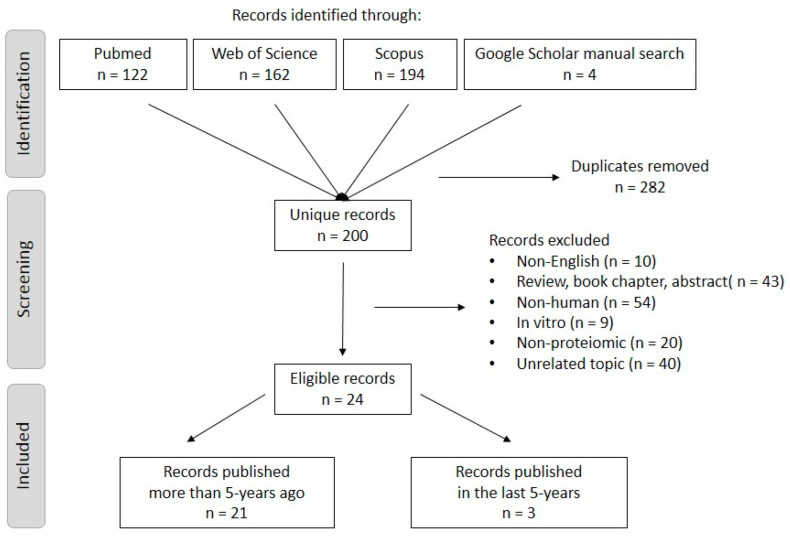
The search process for the PRISMA flow diagram of the included publications.

Our research initially identified 482 published articles: 122 in PubMed, 162 in Web of Science, 194 in Scopus, and 4 were added based on the manual search from Google Scholar. A total of 282 articles were excluded because of duplication and another 176 based on exclusion criteria. Overall, 24 articles went through a full-text review. Only three articles were found to have been published in the last five years, so a time limit was not set for the research. The PRISMA flow chart summarizes the screening process (Figure 1).

We evaluated the selected studies according to the Newcastle–Ottawa Scale (NOS) (Table 2). Based on the assessment, two-thirds of the selected studies demonstrated high quality. Mean ± SD NOS score was 7.16 ± 1.46, suggesting an overall high methodological quality in the studies.

The 24 selected studies were listed in the authors’ alphabetical order with their relevant information for complement component measurement in Table 3.

The sample types used in the selected articles were biofluids, including serum or plasma from blood, sputum, or bronchoalveolar lavage fluid (BALF).

As we did not exclude any article based on its publication date, the measurement methods were heterogenic, and show a large scale of technical variability of protein detection. Forty years of technical revolution from Single Radial Immunodiffusion (SRID) used in the 1980s, through a more sophisticated RIA, ELISA, and LC-MS methods, until the current modern array-based protein profiling techniques has its fingerprint on the way of complement component measurements in asthma.

Regarding the studied population, only one study was population based [63]; however, data from healthy controls were not clearly described in the study. Kay et al. [50] used control samples of those children who were hospitalized with diseases other than asthma. Nakano et al. [59] collected samples from stable asthmatic patients as controls. Subjects recruited to the control groups were age- and sex-matched healthy people in other publications.

We found records about every main complement component, but not within the same article. C1q was measured in two articles. Kay et al. [50] found no significant difference in its concentrations between serum samples of asthmatic patients and controls. Meanwhile Wu et al. [65] found it upregulated in BALF samples from asthmatic patients after allergen challenges. Importantly adding to the issue of sampling from blood or locally from the airways, Kay et al. measured C1q with SRID, while Wu used the MS technique. C2 was only measured by Kay et al. [50], aiming at differentiating allergic and non-allergic asthmatic groups, but finding no difference between them in serum samples. C3 (or its fragments) was measured in 70% of selected articles. C3a was measured from plasma, sputum, and BALF with ELISA, RIA, and Cytometric Bead Array (CBA). Four of five articles found significantly elevated C3a concentrations in asthmatic patients compared to control subjects, both locally in BALF [49,62] and systemically in plasma [53,59], while one article did not find a difference in sputum [55]. Another C3 fragment was measured from serum with SRID in six publications. No difference was found in three publications [45,48,50], while significant elevation was detected in samples from asthmatic patients in three other publications [54,57,58]. C3 level, measured from plasma or serum with immunokits, was significantly higher in samples from asthmatic patients than those of control groups [42,47,51,53]. The MS technique was used in two publications to measure C3 level. Ejaz et al. [46] found a downregulated C3 level, but Rhim et al. found an upregulated C3d level [61]. The study of Vedel-Krogh et al. [63] used the Copenhagen General Population Study consisting of 101,029 individuals with baseline blood levels of C3. They prospectively compared the risk of asthma hospitalization between individuals with the highest and the lowest tertile of blood C3 levels and found that high C3 levels are risk factors for asthma hospitalization in the general population and for exacerbation in allergic asthma. They strengthened their findings by genetic studies and demonstrated a causal role of the complement cascade in asthma. C4 (or its fragments) was measured in 50% of publications. C4a was measured in three publications. Two of them measured C4 and C4a from plasma with the MS technique, and found elevated levels of C4a in immediate-phase reaction [61] and aspirin-induced asthma groups [53]. Marc et al. used CBA on sputum samples but did not find any difference in C4a concentrations between samples obtained from asthmatic patients and control subjects [55]. These complement factors were measured in serum using the SRID method in 42% of publications with variable results. C4 level was found unchanged in two publications [45,58], significantly elevated in the group of asthmatic children in two other publications [50,57], significantly decreased in samples from patients with asthma attacks, but not in stable patients [54] compared to control subjects, respectively. Häfner et al. [48] used serum samples in immunoelectrophoresis, and found elevated C4 levels. The C4 level measured by immunokits from serum was found to be elevated [42] or not changed [45] in samples from asthmatic patients. Another study using MS to measure serum samples found low C4 levels [46]. C5a was significantly higher in sputum [55] and BALF [52] samples from asthmatic patients compared to healthy subjects. It was also elevated in plasma samples of pregnant asthmatic patients compared to healthy pregnant subjects (ELISA) [44]. Membrane attack complex components (C6, C7, C8, C9) were found to be significantly elevated or upregulated in different samples in asthma compared to control groups [43,50,56,65], but the results from different studies were consistent only for the elevation of C9. In cases of C6 and C7 elevation, no changes were found in either [50,60,65], and in the level of C8, one study detected elevation [65], and another one a decrease [46] in samples from asthmatic patients compared to control subjects. Complement cascade regulators (Factor H, Factor I) and Factor B and Factor D were found to be significantly higher or upregulated in asthma [50,51,56,64,65]. In the study of Weiszhár et al., complement Factor H was measured both in induced sputum and plasma samples with no correlation between their concentrations [64]. In this study, sputum Factor H concentrations were higher in samples from asthmatic patients compared to healthy subjects, and correlated with loss of lung function, asthma severity, and medication intensity. A positive correlation was also observed between Factor H/protein ratio and eosinophil cell count in sputum samples in this study [64].

## 4. Discussion

This systematic review addresses the question of whether the complement system is activated in patients with asthma. The different studies provided insights into local changes in the microenvironment of the airways via sputum and bronchoalveolar samples and also into body-wide systemic changes reflected by plasma or serum. The findings indicate that the complement cascade is activated locally and systemically in patients with asthma, as elevated levels of different complement components and regulatory proteins have been detected in both types of biosamples. Elevated level of regulatory proteins, such as Factor H and I, highlight their potential regulatory role in the asthmatic immune responses to different stimuli. The role of complement factors as potential biomarkers of asthma activity and severity and their predictive role in disease worsening needs further confirmation.

The assessment of study quality was conducted using the NOS, a valuable tool for evaluating methodological quality [66], with key components including selection, comparability, and outcome assessment for case–control and cohort studies. However, this tool also has some limitations, including subjectivity and incomplete coverage of study quality dimensions.

Diverse methods, sample origin, and populations made the evaluation of the studies challenging. Differences in used methods (SRID, ELISA, MS) and sample origins (serum, plasma, sputum, BALF) could be the reasons for differences in results. In addition, different results may also be due to different study populations. For instance, Mosca et al. [57] found significantly higher, while Mahdi et al. [54] found significantly lower, C3 levels in asthmatic patients compared to control individuals. Both studies used serum and SRID methods, but Mosca et al. [57] collected serum from Brazilian children, while Mahidi et al. [54] investigated adult male patients in Iraq. Most studies are limited by the relatively small number of study participants. Albeit patients and control subjects were age and sex matched in most studies, they are not representing a larger population well. The study of Vedel-Krogh et al. was based on general population (Copenhagen General Population Study Population) with more than 100,000 participants and demonstrated a causal link of complement cascade activation with asthma severity by demonstrating that higher C3 level is associated with increased risk of asthma hospitalization in the general population [63].

Our current systematic review concluded that despite different sample origins, measurement methods, and populations, a consistent upregulation can be observed in complement system regulator proteins.

The findings of this review indicate that complement pathway regulatory proteins have an impact on asthma. As innate immunity is activated in the first line of host defense, regulatory proteins may serve as potential biomarkers completing existing ones providing a more sophisticated diagnostic and monitoring system.

Several challenges arise regarding blood biomarkers and asthma. It is not well-defined whether peripheral blood effectively represents airway surface and epithelial cells’ local complement activity. Airway sputum and bronchoalveolar lavage fluid would provide more relevant information on the complement and asthma relationship since they represent the airway surface. However, both are difficult samples to handle and interpret the results, as most of the immunokits are optimized for serum or plasma samples and there are some uncertainties regarding the dilution of airway liquid fluid in the samples [67].

Numerous studies have demonstrated the potential effectiveness of the application of monoclonal antibodies that target complement in treating lung diseases [68,69]. The involvement of the complement system in both acute and chronic lung conditions, such as asthma, underscores the opportunity for novel approaches aimed at targeting complement factors to address lung diseases. It seems form the studies covered that complement activation is linked with signaling pathways initiated in type-2 immune reaction, as elevated levels of different complement factors, including C3 and Factor H, are related to eosinophil cell counts [63,64]. Complements however do not seem to be reflected only in this immune pathway, as they cannot fit into any cluster of asthma identified either by protein profiles or clinically relevant subgroups of asthma [56,70].

A deeper understanding and identification of specific phenotypes and endotypes of asthma may lead to the development of tailored therapeutic interventions, potentially improving asthma management and patient care [56,71,72,73,74,75].

Developing precise and effective targeted therapies for asthma may be challenging due to the not fully understand interactions between the complement system and other immune effectors [76]. An improperly timed and targeted blockade of the complement system may open the door to pathogens [77], leading to serious infections.

Based on the current review, there are several key points that need to be further investigated. First, more population-based studies are needed to measure complement components’ concentration in asthmatic patients. Second, these studies could utilize novel and standardized methods for their measurements. It would be valuable to adopt a systemic biological approach to understand more the role of complement system in asthma. Thirdly, subprotein-level studies could also lead to even more precise biomarkers and diagnostic procedures in asthma similarly to that in lung cancer [78].

Continued research into the interplay between innate and adaptive immune responses and the links between other immune host defense mechanisms, including metabolic defenses, such as the activation of purinergic signaling in asthma, is essential for developing more effective prevention and treatment options that target the initial steps of the disease development [79,80,81,82,83]. Novel experimental methods and advanced technologies help us to understand such a complex system such as human immunology and pave the way for groundbreaking advancements in asthma management, thereby fostering enhanced patient outcomes and quality of life globally [79,80].

## 5. Implication of the Findings

So far, there have been numerous attempts to treat asthma. Novel targets, such as inflammatory cytokines, were already targeted with monoclonal antibodies [84], but our findings may offer new ones.

Complement factors, such as biomarkers, may contribute to differentiating subpopulations of asthmatic patients, thus helping to get closer to personalized treatments.

## 6. Limitations

This systematic review has a few limitations. Among the limited number of studies, there were some that were methodologically moderate. Due to the variability of methods and measured proteins, we were unable to perform a meta-analysis, so we instead tried to identify trends among the results.

## 7. Conclusions

Innate immunity plays a pivotal role in the pathogenesis of asthma, influencing airway inflammation, hyperresponsiveness, and exacerbations. This systematic review focuses on the association between asthma and the complement system. Despite discrepancies observed between the studies, we can conclude that complement regulatory proteins are consistently upregulated in asthma. This finding seems to suggest a potential biomarker function for these proteins, complementing current asthma biomarkers, and offering insight into the role of the complement system in disease pathomechanisms. Moreover, this finding suggests a novel predictive tool of asthma exacerbation. Through investigating the interplay between innate immunity and asthma, this review underscores the significance of understanding complement system involvement in the progression of asthma, paving the way for targeted therapeutic interventions and improved patient outcomes.

## Figures and Tables

**Table 1:** The eligibility criteria for the systematic review followed the PICOS domains.

Domain	Inclusion Criteria	Exclusion Criteria
Participants	- Asthmatic patients	- Other respiratory illness
Interventions	- Any intervention - No intervention	
Comparisons	- No control group - Healthy control group - Other respiratory illness	
Outcomes	- Quantitative data of any complement components	- No quantitative data of any complement components - Non proteomic measurement
Study design	- Case–control studies - Cohort studies	- Animal studies - In vitro studies - Systematic reviews - Meta-analyses - Book chapters

**Table 2:** Quality assessment of selected studies in alphabetical order (the NOS tool).

Publication—Author (Year)	Selection	Comparability	Outcome	Total	Quality
Al Mutairi et al. (2011) [42]	3	2	3	8	High
Alabassi et al. (2020) [43]	0	2	3	5	Moderate
Bohács et al. (2016) [44]	4	2	3	9	High
Delaney et al. (1976) [45]	2	1	3	6	Moderate
Ejaz et al. (2018) [46]	0	1	3	4	Moderate
Fattah et al. (2010) [47]	2	1	3	6	Moderate
Häfner et al. (1981) [48]	1	1	3	5	Moderate
Humbles et al. (2000) [49]	2	1	3	6	Moderate
Kay et al. (1974) [50]	1	1	3	5	Moderate
Kirschfink et al. (1993) [51]	3	1	3	7	High
Krug et al. (2001) [52]	4	1	3	8	High
Lee et al. (2006) [53]	3	2	3	8	High
Mahdi et al. (2005) [54]	1	2	3	6	Moderate
Marc et al. (2004) [55]	3	1	3	7	High
Mikus et al. (2022) [56]	3	2	3	8	High
Mosca et al. (2011) [57]	3	2	3	8	High
Najam et al. (2005) [58]	4	2	3	9	High
Nakano et al. (2003) [59]	4	2	3	9	High
Nishioka et al. (2008) [60]	3	2	3	8	High
Rhim et al. (2009) [61]	4	2	3	9	High
van de Graaf et al. (1992) [62]	4	1	3	8	High
Vedel-Krogh et al. (2021) [63]	2	2	3	7	High
Weiszhár et al. (2012) [64]	3	2	3	8	High
Wu et al. (2005) [65]	3	2	3	8	High

**Table 3:** Summary of main results of selected studies.

Publication	Sample	Method	No. Subjects in Asthma	No. Subjects in Control	Complements Investigated in the Publication
Al Mutairi et al. [42]	Blood	immunochemistry	60	42	C3 ↑↑↑, C4 ↑↑↑
Alabassi et al. [43]	Serum	ELISA	30	30	C9 ↑↑↑
Bohács et al. [44]	Plasma	ELISA	41	34	C5a ↑, Factor H ↔
Delaney et al. [45]	Serum	SRID	16	N.A. *^1^	C3 ↔, C4 ↔
Ejaz et al. [46]	Serum	MS	73	99	C3 ↑, C4 ↓, C8 ↓
Fattah et al. [47]	Serum	ELISA	60	60	C3 ↑↑↑, C4 ↔
Häfner et al. [48]	Serum	SRID, “Monorocket” immunoelectrophoresis	159	100	C3 ^‡1^ ↔, C4↑
Humbles et al. [49]	BALF	RIA	8	5	C3a ↑↑↑
Kay et al. [50]	Serum	SRID	93	59 *^2^	C1q ↔, C2 ^‡2^ ↔, C3↔, C4 ^‡3^ ↑↑↑, C6 ^‡4^ ↔, C7 ^‡5^ ↑, Factor B ↑
Kirschfink et al. [51]	Plasma	ELISA	61	30	C3d ↑↑↑, sC5b-9 ↑↑↑, Factor H ↑↑↑, Factor I ↑
Krug et al. [52]	BALF	ABICAP assay	14	9	C3a ↑↑↑, C5a ↑↑↑
Lee et al. [53]	Plasma	MS	54	21	C3 ↑, C3a ^‡6^ ↑↑↑, C4 ↑, C4a ^‡6^ ↑↑↑
Mahdi et al. [54]	Serum	SRID	60	28 *^3^	C3 ↓↓↓, C4 ↓↓↓
Marc et al. [55]	Sputum	CBA	10	12	C3a ↑, C4a ↑, C5a ↑↑↑
Mikus et al. [56]	Plasma	Array-based protein profiling technique	434	91 *^4^	C9 ^‡7^ ↑↑↑, Factor I ^‡7^ ↑↑↑
Mosca et al. [57]	Serum	SRID	40	30 *^5^	C3 ↑↑↑, C4 ↑↑↑
Najam et al. [58]	Serum	SRID	64	57	C3 ^‡8^ ↑↑↑, C4 ↔
Nakano et al. [59]	Plasma	RIA	52	42 *^6^	C3a ↑↑↑
Nishioka et al. [60]	Plasma	ELISA	16	6	C7 ↔
Rhim et al. [61]	Plasma	MS	8	8	C3 ↓, C3d ↑↑↑, C4a ↑↑↑
van de Graaf et al. [62]	BALF, plasma	ELISA, RIA	10	9	C3a ^‡9^ ↑↑↑
Vedel-Krogh et al. [63]	Plasma	Turbidimetry using polyclonal antibodies	2248	101,029 *^7^	C3 *^8^ ↑
Weiszhár et al. [64]	Sputum, plasma	ELISA	26	21	sC5b-9 ↔, Factor H ^‡10^ ↑↑↑
Wu et al. [65]	BALF	MS	4	3	C1q ↑, C6 ↑, C8 ↑, Factor H ↑, Factor B ↑, Factor D ↑

↑↑↑ significantly higher in asthma, ↑ higher in asthma (not significantly), ↔ no difference, ↓↓↓ significantly lower in asthma; ↓ lower in asthma; ELISA—enzyme-linked immunosorbent assay; MS—mass spectrometry; RIA—radioimmunoassay; SRID—single radial immunodiffusion; ABICAP—Antibody Immuno Column for Analytical Processes; CBA—Cytometric Bead Array; *^1^: age-and sex-matched adults attending the general medical clinic were used as controls; *^2^: childhood controls were previously used for clinical purpose; *^3^: non-asthmatic patient, but with sign and symptoms of asthma, were selected and considered as a positive control (*n* = 8); *^4^: only U-BIOPRED cohort data are shown here; *^5^: diagnosis according to the IV Brazilian Asthma Management Directive; *^6^: control subjects were patients with stable chronic asthma; *^7^: the healthy control group data were not provided (only data for the entire Copenhagen General Population Study Population (CGPS) were available); *^8^: the highest and lowest percentile of C3 level were compared with higher levels associated with higher risk of asthma hospitalization in the general population and exacerbation in allergic asthmatic patients; ^‡1^: no difference in mean concentration, significantly higher difference in SD; ^‡2^: method: effective molecular titrations; ^‡3^: significantly higher in children, higher in adults; ^‡4^: method: C6-deficient rabbit serum; ^‡5^: no difference in children, higher in adults; ^‡6^: in aspirin-induced asthma; ^‡7^: in non-smokers with severe asthma; ^‡8^: in active asthma; ^‡9^: significantly higher in BALF measured by ELISA, elevated in plasma measured by RIA; ^‡10^: in sputum.

## Data Availability

The dataset is available on request from the authors.
